# Welfare Work among Boys

**Published:** 1921-03-05

**Authors:** 


### Welfare Work Among Boys.

### AN ABSORBING STORY.

If a modern Plato were to set out to discover which of our social groupings was most valuable to the State he might well decide to give the foremost place to boys' clubs. In these clubs humanity meets on terms that express most of the social virtues. There is freedom, or there are no boys. There is comradeship, for the man who can stand the strain of boys'-club work must have the very essence of that gift. There is mutual help and equality and a mixture of fun and seriousness both at a pitch of intensity which most grown-ups only recapture now and then. There meet in boys' clubs also some of the neediest classes of humanity and some of the most gifted, for the remarkable

**THE PUBLIC WELL-BEING**—(*continued*).

feature of the boys'-club movement is the quality of the young men who have devoted their time and energy to the youngsters of the slums and have done so with an earnestness that most people only reserve for a hobby or a profession. The value of the boys' club to the boy has been enormous. It has given him a friend when he was friendless. It has found a substitute for a father when he would sometimes have been better without his own. It has found him men of education and character to sympathise with troubles and aspirations that few of those about him could understand or cared to take an interest in. It has helped many a boy to rise who did not know how to begin, and it has saved thousands of boys from physical or moral ruin because there has been at hand at critical moments someone with a knowledge of physiology or psychology which the ordinary dweller in the East End can hardly be blamed for lacking.

The boys' club, indeed, the modern Plato would no doubt admit to be one of the finest products of a civilised State. It has led all along the line to improvements in the social conditions of boyhood. Barristers, clergymen, employers, and local administrators have learned in boys' clubs of the defects of laws and regulations and have been moved to amend them. Lately, however, the boys'-club movement has earned a still higher claim to merit, for out of it has grown the practice in large firms of appointing boys' welfare supervisors whose existence will have the most profound effects on the boy in industry and on industry itself. Just as the introduction of the doctor into school and factory has had the most far-reaching effects on both, so the existence inside a factory of a man looking at things primarily from the boy's point of view will react on the whole conduct of industry for good. How great is the difference such an appointment will make to the individual boy is shown by the Rev. R. R. Hyde in his new book "The Boy in Industry and Leisure" (Bell, 6s.). Mr. Hyde tells an absorbing story of his work in Hoxton among the young hopefuls of the East End, but the most important part of his work was two years spent at the Ministry of Munitions in getting firms to care for the welfare of their boy employees, and the work to which he has more lately devoted himself of organising a voluntary society, controlled by masters and men, for the spreading of welfare work. He shows how much it is needed and how much it can do by pointing out that the working-class boy is thrown at the most critical moment of his life into a job for which his physical fitness has not been considered, where at a bound he changes from a child to a man among men, subject from the very first day to their hours and conditions, and with less than a man's power to claim his rights or explain his grievances. Some painful instances are quoted of most promising boys ruined by the unconscious injustice of their superiors.

In a works where a welfare adviser is engaged he receives all the new boys, and has time, as a foreman would never have, to explain to them the new conditions they are entering on, to give them warning and advice and to offer them a channel for seeking information or securing help. Under an efficient supervisor the medical inspection of boys is usually carried out under circumstances of the greatest difficulty for the doctor, since boys are rushed out from work with a warning to hurry back again; this can be immensely improved by having the boys ready for him and by a hint offered as to cases in which a short examination would fail to reveal real defects. The doctor will be helped by knowing that his advice will be really understood and that there is someone interested in carrying it out. These are only instances of the ways in which welfare work can benefit both the employer and the boy. The movement is bound to have the most far-reaching effects, as all the best employers have recognised, and as some of the others are being compelled to recognise, because the firms with welfare systems get the best boys. It is a pity that Mr. Hyde's book does not contain a chapter on welfare work for girls. It is an occupation of profound interest to all members of the nursing profession who realise the growing social importance of their work.

